# Crystallographic and electronic properties of AlCrN films that absorb visible light

**DOI:** 10.1063/1.4983491

**Published:** 2017-05-10

**Authors:** N. Tatemizo, S. Imada, Y. Miura, K. Nishio, T. Isshiki

**Affiliations:** Faculty of Electrical Engineering and Electronics, Kyoto Institute of Technology, Kyoto 606-8585, Japan

## Abstract

We investigate the crystallographic and electronic properties of wurtzite Cr-doped AlN (AlCrN) films (Cr ≤12.0%) that absorb visible light. We confirmed that the films consist of wurtzite columnar single crystals that are densely packed, *c*-axis oriented, and exhibit a random rotation along the *a*-axis in plane by using transmission electron microscopy. The oxidation state of Cr was found to be 3+ using Cr *K*-edge X-ray absorption near edge structure, which implies that Cr can be a substitute for Al^3+^ in AlN. The first nearest neighbor distances estimated using Cr *K*-edge extended X-ray absorption fine structure (EXAFS) were found to be nearly isotropic for incident light with electric fields that are parallel and perpendicular to the plane. The results of *ab initio* lattice relaxation calculations for the model of wurtzite Al_1-x_Cr_x_N supercell where Cr replaces Al support the EXAFS results. The calculations for the model showed that additional energy bands are formed in the band gap of AlN, in which the Fermi energy (*E*_*F*_) is present. As expected from the calculation results, the electrical conductivity increases with increase in the Cr concentration, implying that the density of states at *E*_*F*_ increases monotonically. From these results, we can conclude that AlCrN films are an intermediate band material with respect to their crystallographic and electric properties.

AlN is a group-III nitride that has been extensively studied.^1^ Alloys of AlN, GaN, and InN can be used to fabricate light emitting diodes and laser diodes that operate in the infrared to ultraviolet regions through band gap engineering.^2,3^ Alloys of GaN and InN are also promising candidates for solar cells, when modified via the same technique.^4–6^ However, AlN cannot be used for photovoltaics because of its large band gap of 6.2 eV.^1^

Intermediate band (IB) materials have been proposed for use in absorption layers of solar cells by Luque *et al.* to increase their conversion efficiency.^7–9^ These materials are characterized by the existence of partially filled energy bands within the intrinsic band gap of semiconductors that split the intrinsic band gap into sub-band gaps. Consequently, mobile holes and electrons are generated in the intrinsic valence and conduction bands, respectively, at an energy that is less than the intrinsic band gap energy *E*_*g*_. This band structure engineering approach of IB can be applied to AlN in order to make it suitable for photovoltaics; however, to date this approach has been applied only to semiconductors with *E*_*g*_ in the visible- infrared light region.^10–14^

It is known that 3*d*-transition metals (3*d*-TMs) can form impurity levels in the band gap of a semiconductor, and these levels have the ability to accept and/or donate electrons when irradiated with light of appropriate energy.^15^ When the concentration of 3*d*-TMs is sufficiently high, it is expected that bands would be formed.^8,9,11^ Based on this hypothesis, we have synthesized and investigated Cr-doped AlN (AlCrN) films. In our previous study,^16^ it is shown that electron-occupied Cr 3*d* states are formed at the top of the valence band and in the band gap of AlN. This implies that Cr ions are surrounded by N ions, and form bonding and antibonding states. In this study, we analyze the crystallographic structures and charge transport properties of AlCrN films.

AlCrN films were deposited on SiO_2_ glass substrates by radio-frequency sputtering. The details of the sample preparation are described elsewhere.^16^ All the films discussed in this paper show optical absorption in the ultraviolet-visible-infrared light region (see Fig. S1 of the supplementary material). X-ray diffraction (XRD) measurements confirmed that the films have *c*-axis-oriented wurtzite structure.^16^ Samples for transmission electron microscopy (TEM) were prepared by mechanical polishing followed by ion-thinning methods. Cross-sectional and plan-view TEM images were obtained using a JEOL JEM-2010 at 200 kV. The 3*d*-TM *K*-edge X-ray absorption fine structure measurements were performed at the BL9A^17^ and BL12C^18^ beam lines of the Photon Factory at the High Energy Accelerator Research Organization in Tsukuba, Japan in fluorescence-detection mode using an array of 19 elements of Ge solid-state detectors. The electric field vectors of the X-rays were at angles of 8° and 84° with respect to the film plane and were approximately perpendicular and parallel to the *c*-axis of the wurtzite films, respectively. The electrical DC properties of the films were studied using Ni/Au electrodes (0.5 mm × 1.0 mm with 0.5 mm spacing) deposited on the films. All measurements were performed at room temperature (RT).

The relaxation of atomic positions and the total density of states (TDOS) of Al_1-*x*_Cr_*x*_N were calculated with first-principles density functional calculations using the Vienna *ab initio* simulation package.^19,20^ The calculation details are described in our previous report.^16^ In this study, the lattice constants of the AlCrN films evaluated from the XRD measurements were applied for periodic supercells. We considered Cr concentrations between x = 0.00 (Al_36_N_36_) and x = 0.111 (Al_32_Cr_4_N_36_), in which some of the host Al atoms were replaced by a Cr atom. Dispersed and clustering models were applied for supercells of Al_32_Cr_4_N_36_.

Figure 1(a) shows the cross-sectional dark-field TEM image of the AlCrN (Cr: 12.0%) film with the 0002 diffraction spot of the wurtzite AlN structure. It is observed that the film consists of columnar crystals with ∼100 nm diameter, and there is no indication of voids between the columns. Figure. 1(b) shows the selected area electron diffraction (SAED) pattern of the area marked with a circle in Fig. 1(a), taken along the [11-20] zone-axis of the wurtzite structure. This reveals that the columnar grains are wurtzite single crystals with a *c*-axis preferred orientation in accord with the XRD results. This conclusion is further supported by the plan-view high-resolution TEM results shown in Fig. 1(c) and the inset SAED pattern of the center grain. The SAED pattern indicates a clear [0002] diffraction in the wurtzite structure, which means that the grain is a single crystal with a *c*-axis preferred orientation. No preferred orientation of the *a*-axis was observed for the in-plane orientation (Fig. 1(d)), presumably owing to the glassy nature of the SiO_2_ substrate.

**FIG. 1.**
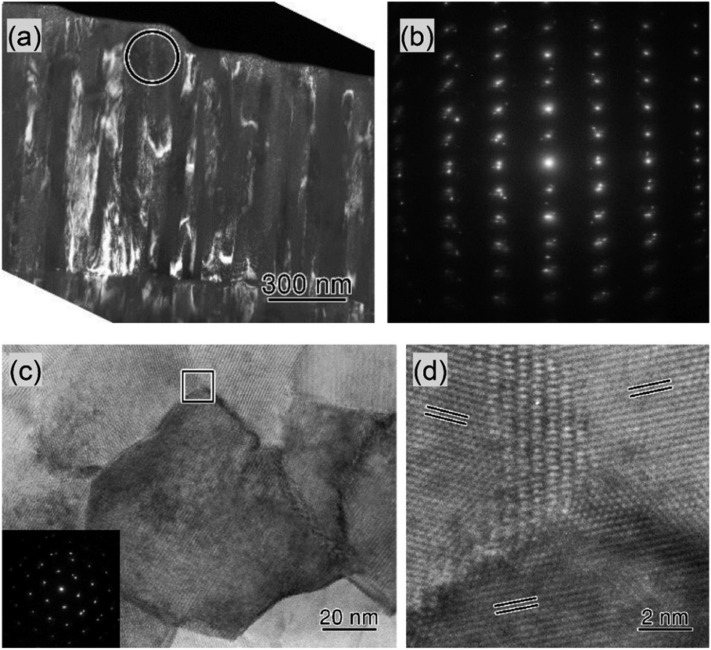
(a) Cross-sectional 0002 dark-field TEM image of AlCrN (Cr: 12%) film and (b) corresponding [11-20] SAED pattern of the area marked by the circle in (a). (c) Plan-view high-resolution TEM image. The inset is [0002] SAED pattern of the center grain. (d) Enlarged TEM image at a triple junction corresponding to the box marked in (c) for an as-deposited AlCrN (Cr: 12%) film grown on a SiO_2_ substrate. The double lines in (d) correspond to the {1-100} plane.

The oxidation states of Cr were deduced by comparing the measurements of Cr *K*-edge X-ray absorption near edge structure (XANES) for the film and for standard materials such as Cr metals, Cr_2_O_3_, and CrN. Fig. 2(a) shows the normalized Cr *K*-edge XANES spectra of AlCrN (12.0%), Cr metal (formal valence of Cr = 0) foil, CrN (3+) powder, and Cr_2_O_3_ (3+) powder. The main absorption edges of Cr in the AlCrN film are close to that of Cr in CrN (3+) and Cr_2_O_3_ powder, and away from those of the Cr metal (0). This implies that Cr in the AlCrN films is nearly trivalent, which is valid when Cr as a substitute for Al (3+) in AlN. The main absorption edge energies do not show a dependency on the Cr concentration (see Fig. S2 of the supplementary material), and imply that Cr can be substituted for Al in AlN in the concentration range. Pre-edge absorption peaks are clearly observed in the spectra of AlCrN films, whereas they are not well resolved in CrN and Cr_2_O_3_. This indicates that the Cr in AlN is surrounded by N atoms with non-centrosymmetric conditions such as *C*_3*v*_, *D*_*2d*_, and *T*_*d*_, different from the Cr in CrN and in Cr_2_O_3_, which are coordinated with six anions with a center of inversion.^21–23^

**FIG. 2.**
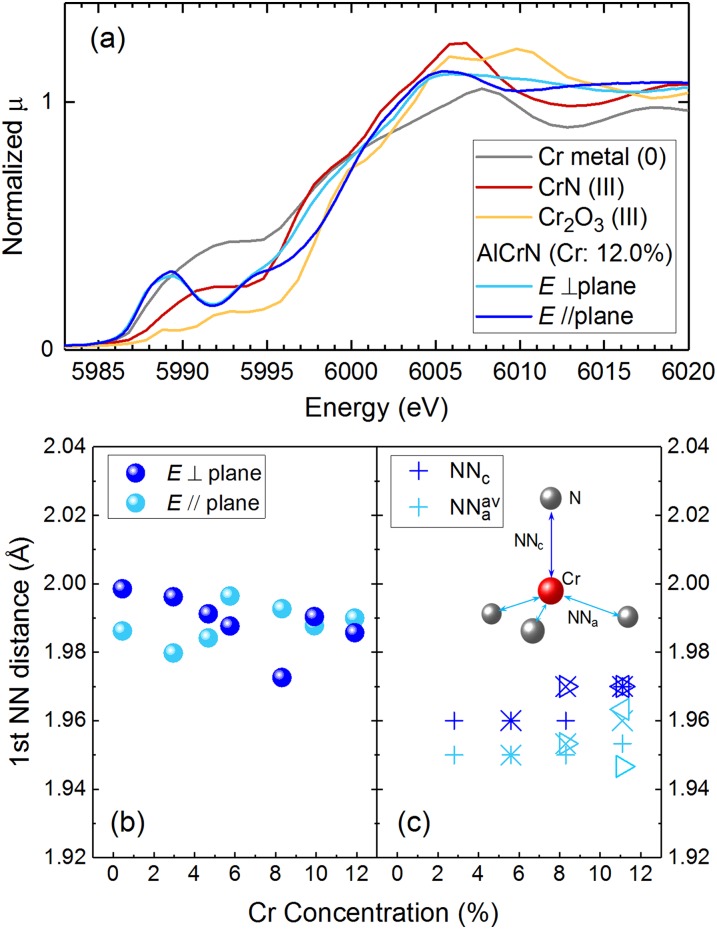
(a) XANES spectra of AlCrN (Cr: 12%) with Cr metal, CrN, and Cr_2_O_3_. These spectra are normalized with their signal intensities in the EXAFS region. (b) The first nearest neighbor (NN) distances for Cr in E⊥ plane and *E* // plane modes plotted against the Cr concentration. (c) The calculated first NN distances for Cr. NN_C_ is the distance to an N atom parallel to the c-axis of wurtzite structure. NN_a_^av^ is the average distance to the other three N atoms.

Next, the Cr *K*-edge extended X-ray absorption fine structure (EXAFS) spectra were analyzed. From the oscillatory portion of the spectra *χ*(*k*) in *k*^3^ weighted form, the first nearest neighbor (NN) region contributions were isolated as *k*^3^ weighted *χ*(*q*), and fitted for all the samples by assuming that Cr substitutes for Al in AlN. Wurtzite AlN structures with the target atoms Cr occupying Al sites were constructed as fitting models, and single scattering spectra with scattering paths from the Cr to the nearest N atoms were calculated using the FEFF6 code.^24^ The lattice constants estimated via XRD measurements were used in the models. The experimental *χ*(*q*) were in good agreement with the theoretical values (see Fig. S3 of the supplementary material). The best-fit Cr-N distances were extracted and plotted against the Cr concentrations for the E⊥ plane and *E*// plane modes as shown in Fig. 2(b). No clear dependence on the Cr concentration is observed up to 12%. The bond-length differences in the two directions are similar, which implies that Cr could have *D*_*2d*_ or *T*_*d*_ site symmetry.

Similar features are observed from Fig. 2(c), which shows the theoretical bond-length obtained using lattice relaxation calculation for the dispersed model. The theoretical first NN distances, NN_c_ and NN_a_^av^, correspond to those estimated via EXAFS in *E*// plane and E⊥ plane modes, respectively. The difference between the bond-lengths NN_c_ and NN_a_^av^ (average value of the three bonds) is small, and the same as that obtained using EXAFS. Moreover, there is no obvious dependence on the Cr concentration. The center values are slightly different, ∼1.98 Å from EXAFS, and ∼1.96 Å from the theoretical results. This could be attributed to the difference in the temperature—the EXAFS measurements were carried out at RT, while a temperature of 0 K was assumed for the theoretical computations.

It is worth noting that when some Cr atoms substitute close Al sites, for example, a Cr-N-Cr cluster, the first NN distances are different from that of the dispersed model. For example, the values range from 1.88 to 2.02 Å for the Al_32_Cr_4_N_36_ model when three out of four Cr atoms form a cluster, while they range from 1.94 to 1.97 Å for the dispersed model. The possibility of the formation of such a cluster increases with the Cr concentration stochastically. The cluster formation changes the crystallographic as well as the electric structure. In fact, the shape of bands in the energy gap for the clustering model is quite different from that in the dispersed model as shown in Fig. 3; the bands split into more than three due to a low symmetric local structure of Cr. As the spectra of the optical absorption, the Cr *K*-edge XANES, and the X-ray photoemission^16^ of the films showed no structural changes for different Cr concentrations, it is thought that Cr substitutes Al in a well-dispersed manner even at high Cr concentrations of 12%.

**FIG. 3.**
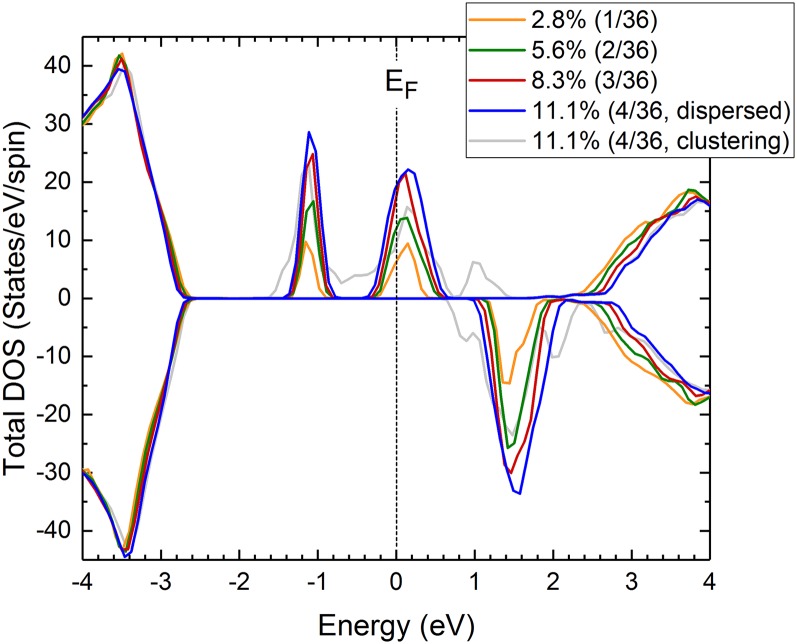
Theoretical spin-polarized total DOS for AlCrN with 2.8 (Al_35_Cr_1_N_36_), 5.6 (Al_34_Cr_2_N_36_), 8.3 (Al_33_Cr_3_N_36_), and 11.1% (Al_32_Cr_4_N_36_, for the dispersed and the clustering models) of Cr.

The TDOS at the *E*_*F*_ increases monotonically with increase in the Cr concentration for well-dispersed models, as shown in Fig. 3. In this case, it is expected that electric conductivity will increase with Cr concentration, if appropriate metal electrodes are used for measurement. The electrodes must have sufficient work function to draw carriers out from the band in the band gap. We carried out I–V measurements with Ni/Au electrodes. The work function of Ni is 5.15 eV,^25^ which is close to the highest occupied electron states in the AlCrN films estimated using photoelectron yield spectroscopy.^16^ The I–V curve of a film with 8.6% Cr is plotted in the inset of Fig. 4 as an example. It shows a linear response, meaning that the Ni electrodes are in contact with the band in AlCrN. The main panel of Fig. 4 shows the Cr concentration dependence of the sheet conductivity of the AlCrN films. The sheet conductivity increases with increase in the Cr concentration, implying that the TDOS at *E*_*F*_ increases. However, the effects of the columnar structure on the charge transport are not clear. For single band gap materials, such as GaN, double Schottky junctions are thought to be formed at the interface of the columns.^26^ For the AlCrN films on glass substrates, the average of the diameters of the columns is almost the same in this Cr concentration range. Hence, we conclude that the changes in the sheet conductivity reflect the Cr concentration.

**FIG. 4.**
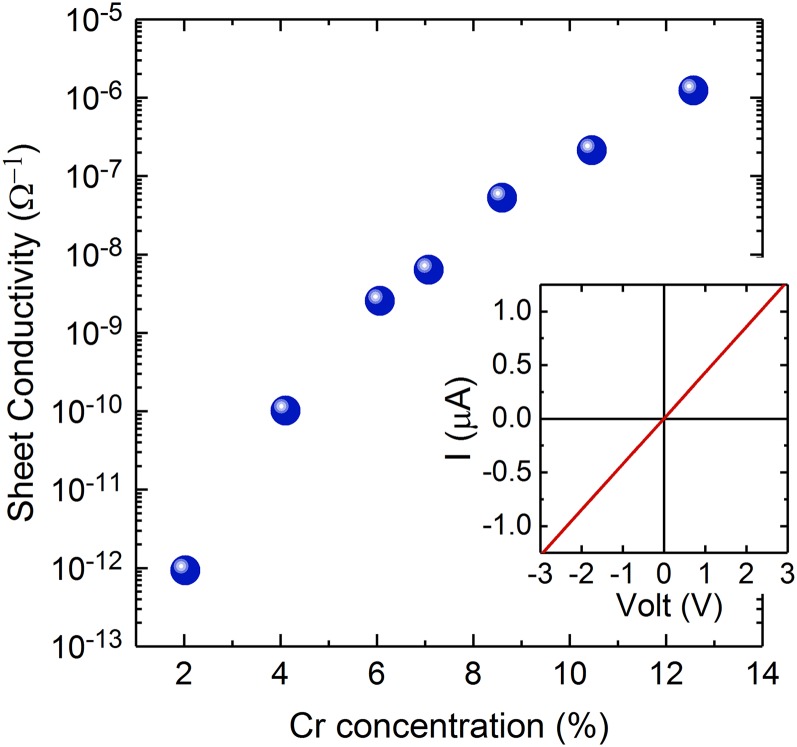
Cr concentration dependence of the sheet conductivity of the AlCrN films. The inset is an I–V curve for an AlCrN film with 8.6% of Cr.

We plan to use the time-dependent density functional theory method to investigate the optical absorption, as this method can correctly describe the excitation of an electron from the valence states. In addition, to investigate if the expected multistep photoexcitation occurs, we will conduct both transient absorption measurements and photoconductivity measurements under multi-wavelength light irradiation for the films.

In summary, we synthesized AlCrN films and studied their crystallographic and electronic properties. TEM measurements revealed that the AlCrN films consist of *c*-axis-oriented wurtzite columnar single crystals. The XANES spectra indicated that Cr ions have an oxidation state of 3+, resulting in their possible substitution for Al^3+^ in AlN. The EXAFS analysis showed that the first NN distances were nearly isotropic. The electrical conductivity increased with increase in the Cr concentration. The *ab initio* lattice relaxation and TDOS calculations supported these results. From these results with our previous study for the electronic structure of the films,^16^ we recommend the AlCrN films as a potential IB material for efficient photoconversion.

## SUPPLEMENTARY MATERIAL

See supplementary material for the optical absorption coefficient α spectra of AlCrN films (FIG. S1), the Cr *K*-edge XANES spectra for various Cr concentrations (FIG. S2), and real part of experimental *k*^*3*^-weighted χ(*q*) and best fit curve of theoretical values (FIG. S3).

## References

[1] S. Strite, J. Vac. Sci. Technol. B Microelectron. Nanom. Struct. 10, 1237 (1992). 10.1116/1.585897

[2] K. Balakrishnan, T. Katona, and A. Khan, Nat. Photonics 2, 77 (2008). 10.1038/nphoton.2007.293

[3] Y. Taniyasu, M. Kasu, and T. Makimoto, Nature 441, 325 (2006). 10.1038/nature04760 16710416

[4] R. Dahal, B. Pantha, J. Li, J. Y. Lin, and H. X. Jiang, Appl. Phys. Lett. 94, 063505 (2009). 10.1063/1.3081123

[5] E. Matioli, C. Neufeld, M. Iza, S. C. Cruz, A. A. Al-Heji, X. Chen, R. M. Farrell, S. Keller, S. DenBaars, U. Mishra, S. Nakamura, J. Speck, and C. Weisbuch, Appl. Phys. Lett. 98, 021102 (2011). 10.1063/1.3540501

[6] A. Mukhtarova, S. Valdueza-Felip, L. Redaelli, C. Durand, C. Bougerol, E. Monroy, and J. Eymery, Appl. Phys. Lett. 108, 161907 (2016). 10.1063/1.4947445

[7] A. Luque and A. Martí, Phys. Rev. Lett. 78, 5014 (1997). 10.1103/physrevlett.78.5014

[8] A. Luque, A. Martí, and C. Stanley, Nat. Photonics 6, 1 (2012).

[9] A. Luque and A. Martí, Adv. Mater. 22, 160 (2010). 10.1002/adma.200902388 20217682

[10] Y. Okada, N. J. Ekins-Daukes, T. Kita, R. Tamaki, M. Yoshida, A. Pusch, O. Hess, C. C. Phillips, D. J. Farrell, K. Yoshida, N. Ahsan, Y. Shoji, T. Sogabe, and J.-F. Guillemoles, Appl. Phys. Rev. 2, 21302 (2015). 10.1063/1.4916561

[11] P. Olsson, C. Domain, and J. F. Guillemoles, Phys. Rev. Lett. 102, 1 (2009). 10.1103/PhysRevLett.102.227204 19658900

[12] C. Tablero, A. Martí, and A. Luque, J Appl Phys 105, 33704 (2009). 10.1063/1.3074311

[13] A. Boronat, S. Silvestre, D. Fuertes Marrón, L. Castañer, A. Martí, and A. Luque, J. Mater. Sci. Mater. Electron. 24, 993 (2013). 10.1007/s10854-012-0864-9

[14] A. Martí, D. F. Marrón, and A. Luque, J. Appl. Phys. 103, 073706 (2008). 10.1063/1.2901213

[15] U. Gerstmann, A. T. Blumenau, and H. Overhof, Phys. Rev. B 63, 75204 (2001). 10.1103/physrevb.63.075204

[16] N. Tatemizo, S. Imada, Y. Miura, H. Yamane, and K. Tanaka, J. Phys. Condens. Matter 29, 85502 (2017). 10.1088/1361-648x/aa5381 28081007

[17] M. Nomura, J. Synchrotron Rad 6, 182 (1999). 10.1107/s0909049598016823 15263241

[18] M. Nomura and A. Koyama, KEK Rep. 95-15(27), 1 (1996).

[19] G. Kresse and J. Hafner, Phys. Rev. B 47, 558 (1993). 10.1103/physrevb.47.558 10004490

[20] J. P. Perdew, K. Burke, and M. Ernzerhof, Phys. Rev. Lett. 77, 3865 (1996). 10.1103/physrevlett.77.3865 10062328

[21] I. Quasicrystals 71, 4166 (1993). 10.1103/PhysRevLett.71.4166 10055173

[22] X. Y. Zhang and D. Gall, Phys. Rev. B 82, 45116 (2010). 10.1103/physrevb.82.045116

[23] K. E. Miyano, J. C. Woicik, P. S. Devi, and H. D. Gafney, Appl. Phys. Lett. 71, 1168 (1997). 10.1063/1.119615

[24] B. Ravel and M. Newville, J. Synchrotron Radiat. 12, 537 (2005). 10.1107/s0909049505012719 15968136

[25] H. B. Michaelson, J. Appl. Phys. 48, 4729 (1977). 10.1063/1.323539

[26] L. Sw, U. K. Several, T. Cc, T. A. B. Le, A. Z. Bi, M. C. Na, and E. Cc 14, 676 (1995).

